# Lambert‐Eaton myasthenic syndrome with primary thymic marginal zone B‐cell lymphoma: A case report

**DOI:** 10.1002/rcr2.1149

**Published:** 2023-04-18

**Authors:** Toshihiko Soma, Akira Matsumoto, Tsuyoshi Shoji, Hiromichi Katakura

**Affiliations:** ^1^ Department of Thoracic Surgery Otsu Red Cross Hospital Otsu‐shi Japan

**Keywords:** Lambert‐Eaton myasthenic syndrome, mediastinal tumour, multidisciplinary treatments, primary thymic marginal zone B‐cell lymphoma, robot‐assisted extended thymectomy

## Abstract

Lambert‐Eaton myasthenic syndrome (LEMS) is an autoimmune paraneoplastic syndrome with proximal muscle weakness, that often complicates small cell lung cancer. It is known that neurological symptoms do not improve with malignancy treatment alone in many LEMS patients, therefore treatment is often difficult. Since Lambert‐Eaton myasthenic syndrome is a rare disease with a frequency of about 1/100 that of myasthenia gravis, there are only a few case reports on malignancy complications other than small cell lung cancer. We report a LEMS patient in his 40s who was found to have an anterior mediastinal mass. We performed surgical resection and confirmed the diagnosis of primary thymic marginal zone B‐cell lymphoma by pathological diagnosis using immunostaining. Thymectomy and malignant lymphoma treatment with rituximab had no effect on neurological symptoms. The neurological symptoms improved only after we provided comprehensive care with the haematology, neurology, and rehabilitation department.

## Graphical abstract

Lambert‐Eaton myasthenic syndrome (LEMS) is a condition in which antibodies are produced against voltage‐gated calcium channels in presynaptic nerve endings, resulting in an inability to release acetylcholine. It is characterized by fatigue and weakness of the proximal muscles of the extremities and is complicated by ocular symptoms, autonomic symptoms, and cerebellar ataxia. We report the case of a patient in whom comprehensive care with multiple departments successfully improved LEMS symptoms with primary thymic marginal zone B‐cell lymphoma.

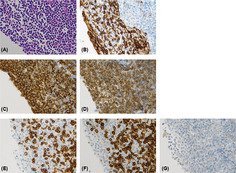

## INTRODUCTION

Lambert‐Eaton myasthenic syndrome (LEMS) is a condition in which antibodies are produced against voltage‐gated calcium channels in presynaptic nerve endings, resulting in an inability to release acetylcholine. It is characterized by fatigue and weakness of the proximal muscles of the extremities and is complicated by ocular symptoms, autonomic symptoms, and cerebellar ataxia. Small cell lung cancer (SCLC) is the most commonly associated tumour in patients with LEMS and it is essential to search for malignant tumours. However, it is known that neurological symptoms do not improve with malignancy treatment alone in many LEMS patients, therefore treatment is often difficult. Since LEMS is a rare disease with a frequency of about 1/100 that of myasthenia gravis, there are only a few case reports on malignancy complications other than SCLC. We experienced a case that comprehensive care with multiple departments successfully improved LEMS symptoms with primary thymic marginal zone B‐cell lymphoma.

## CASE REPORT

**FIGURE 1:**
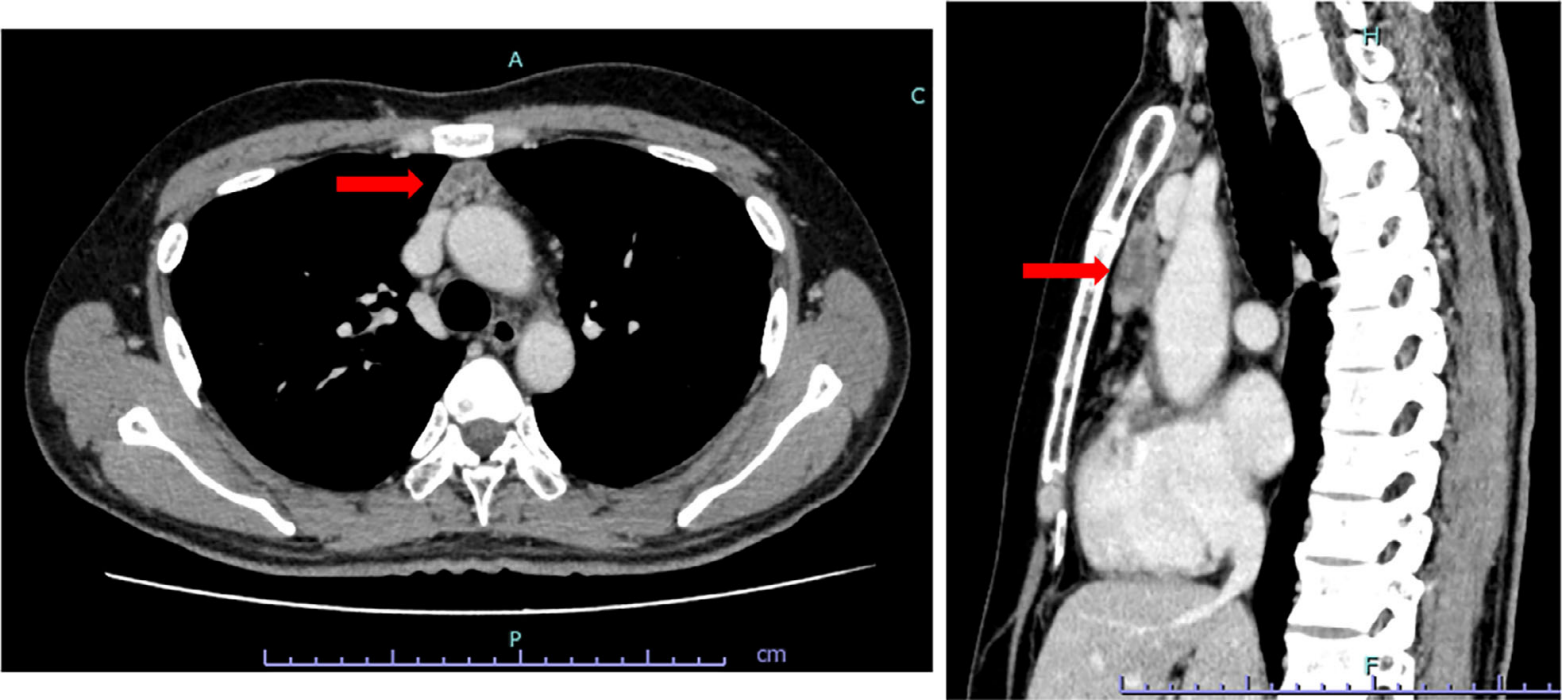
Contrast‐enhanced computed tomography revealed a 4.0 × 2.5 cm mass in the anterior mediastinum with multiple cysts inside, suggesting thymoma, thymic hyperplasia, or lymphoma.

A man in his 40s was presented to the neurology department at our hospital due to the gradual worsening of weakness in both lower limbs since 6 months ago. His past medical history included Perthes disease (12 years old), systemic lupus erythematosus (SLE) (16–20 years old), and Sjogren's syndrome. He was smoking 20 cigarettes a day and taking no medication. Although there was no decrease in sensory‐tactile sensation or decrease in warmth‐pain sensation, he had a positive Gowers' sign, symmetrical lower limb weakness, and was unsteady when standing on one leg. The nerve conduction test showed a marked improvement in the amplitude of compound muscle action potential after exercise. P/Q‐type voltage‐gated calcium channel antibody in serum was high (234.5 pmol/L), and the neurologists diagnosed him with LEMS. Elevated autoantibodies consistent with pre‐existing Sjogren's syndrome were also found (anti‐SSA/Ro antibodies 5990 U/mL and anti‐SSB/La antibodies 2320 U/mL). Contrast‐enhanced Computed tomography scan of the chest (Figure 1) revealed a 4.0 × 2.5 cm mass in the anterior mediastinum with multiple cysts inside, suggesting thymoma, thymic hyperplasia, or lymphoma. Fluorodeoxyglucose‐positron emission tomography (FDG‐PET) showed moderate FDG accumulation in the anterior mediastinal mass and mild FDG accumulation in the bilateral axillary lymph nodes.

The patient was referred to the thoracic surgery department for the diagnosis and treatment of the anterior mediastinal mass complicated by neurological symptoms. Considering the possibility of thymoma, we decided to perform surgical resection without biopsy because of the risk of the mediastinum, chest wall, and needle tract dissemination. A robot‐assisted extended thymectomy was performed for the anterior mediastinal mass.

**FIGURE 2:**
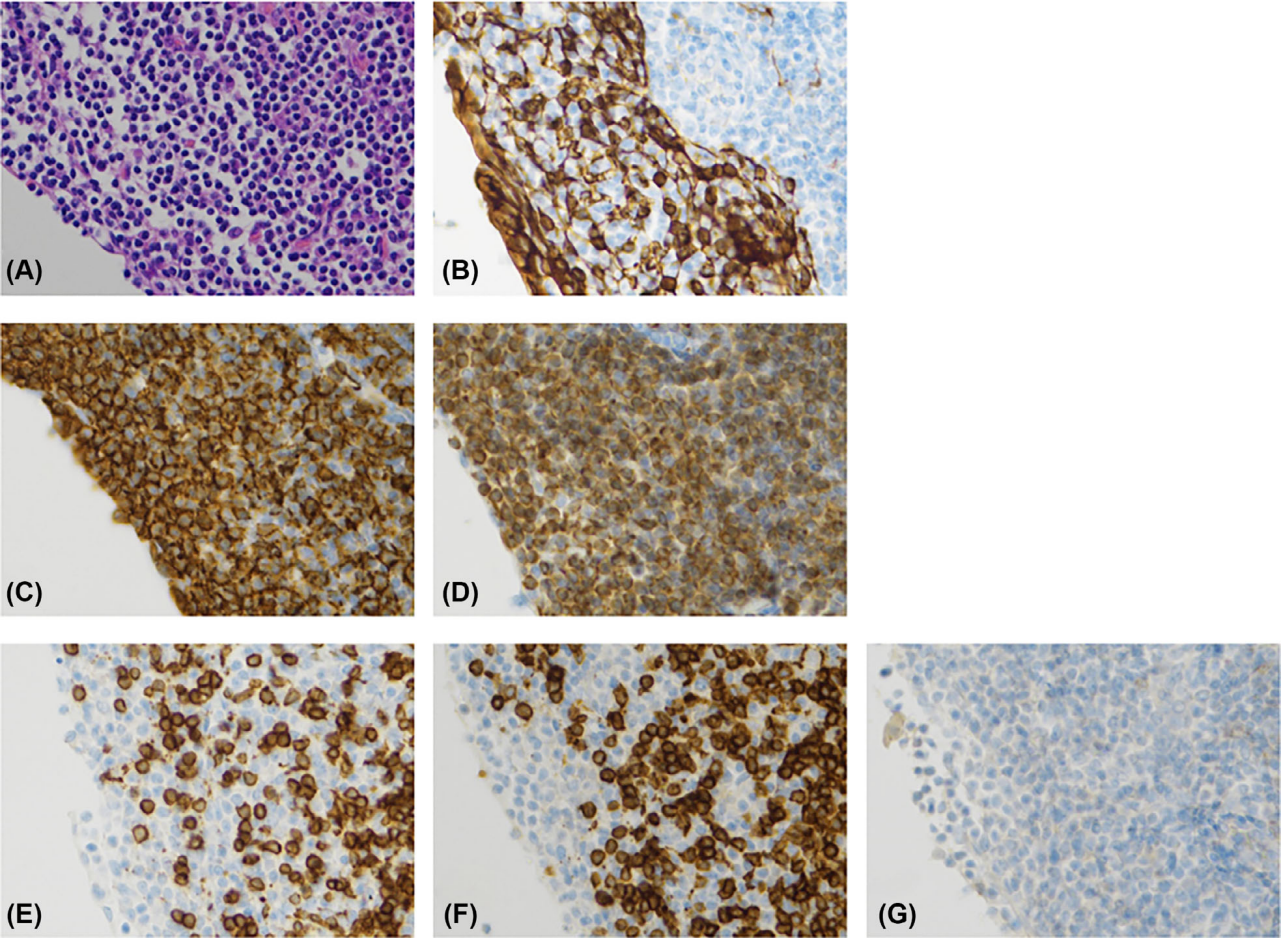
200×, at the same field. Cytokeratin(AE1/AE3)‐positive thymic epithelium were invaded by the lymphocytes positive for CD20 and bcl‐2, but negative for CD3, CD5, and CD10. (A) HE, 200×. (B) Cytokeratin(AE1/AE3) positive. (C) CD20 positive. (D) bcl‐2 positive. (E) CD3 negative. (F) CD5 negative. (G) CD10 negative

Grossly, the anterior mediastinal mass was a yellow surface measuring 13 cm in dimension. The cut surface showed fleshy, light tan to brown, multinodular lesions with scattered small cysts. Microscopically, the cysts containing proteinaceous material were covered by thymic epithelial cells. The cyst wall contained small lymphocytes, plasma cells, and lymphoid follicles. The lymphocytes were positive for CD20 and bcl‐2, but negative for CD3, CD5, and CD10 (Figure 2). The lymphocytes invaded Hassall bodies and thymic epithelial cells, forming lymphoepithelial lesions. Those findings have led to the diagnosis of primary thymic extranodal marginal‐zone B‐cell lymphoma of mucosa‐associated lymphoid tissue type.

The postoperative course was uneventful and the patient was discharged on the 11th day after the surgery. One month after the surgery, he was referred to the haematology department and treated with 700 mg rituximab three times for lymphoma, however, his LEMS symptoms did not improve much. Two months after the surgery, the patient was transferred to the neurology department and treated with a three‐day steroid pulse using 1000 mg methylprednisolone per day, 15 mg diaminopyridine, five‐day intravenous immune globulin with 5 units per day, and 50 mg azathioprine. Rehabilitation was also performed and his symptoms improved gradually, and the nerve conduction test showed improvement as well. Annual CT scan shows no recurrence of thymic lesions or appearance of lung cancer. He is currently receiving 60 mg diaminopyridine and 100 mg azathioprine as an outpatient.

## DISCUSSION

It is known that about half of LEMS patients have SCLC. In a clinical series of 50 patients with LEMS, 25 patients had concomitant malignancies, and 21 of them (84%) had small cell lung cancer. The incidence and prevalence of LEMS in patients with small cell lung cancer are about 3% each.^1^ SCLC is often detected within 2 years after the onset of LEMS. In this patient, no images were suggesting the development of SCLC within 2 years after the onset of LEMS, and there was no elevation of tumour markers. Some case reports have shown that lymphoproliferative disorders and autoimmune diseases are associated with LEMS, and our patient also had malignant lymphoma and a history of SLE and Sjogren's syndrome.^2^, ^3^

In this case, the finding of CD20 and bcl‐2 positive lymphocytes invading Hassall bodies and thymic epithelial cells led to the definitive diagnosis of primary thymic extranodal marginal‐zone B‐cell lymphoma of mucosa‐associated lymphoid tissue type. Isaacson was the first to report two cases of primary thymic marginal zone B‐cell lymphoma in 1990, and there have been sporadic reports since then. Many cases are associated with autoimmune diseases, with Sjögren's syndrome being the most frequently reported.^4^

Tim et al. reported that malignancy treatment alone improved LEMS symptoms in only 9 out of 26 patients.^5^ Commonly, malignancy treatments alone do not improve the neurological symptoms in LEMS patients. Thymectomy and malignant lymphoma treatment with rituximab were insufficient to improve neurological symptoms in this case as well. We have succeeded in treating LEMS symptoms by providing comprehensive patient care with the cooperation of the haematology, neurology, and rehabilitation departments. Therefore, we believe that patients with LEMS require multidisciplinary treatments in which multiple departments cooperate to improve symptoms.

## AUTHOR CONTRIBUTIONS

Toshihiko Soma and Tsuyoshi Shoji wrote the manuscript, which was then reviewed by all co‐authors.

## CONFLICT OF INTEREST STATEMENT

None declared.

## ETHICS STATEMENT

The authors declare that appropriate written informed consent was obtained for the publication of this manuscript and accompanying images.

## Data Availability

Data available on request due to privacy/ethical restrictions
